# Editorial

**Published:** 1839-08-10

**Authors:** 


### THE MEDICAL EXAMINER.

PHILADELPHIA, AUGUST 10, 1839.

One of our correspondents furnishes us with an analysis of the debates of the Royal Academy of Medicine, of Paris, on the subject of the distinction between the nerves of motion and of sensation. These debates are almost interminable, and occupy a large portion of the late numbers of the Bulletin of the Academy of Medicine. Like the debates of most learned societies, they have ended by leaving every one precisely of the same opinion which he held at the time they began;—we ought not, on that account, to conclude that they have been altogether fruitless. Arguments in medicine, as in every thing else, do not convince, but they often awaken reflection, and give rise to new and more exact observations, and thus indirectly elicit the truth.

The composition of the Royal Academy is like that of most other scientific societies,—it consists of men who have attained distinction by glorious and successful contributions to science, and of others who are admitted to a seat on the strength of a doubtful and almost surreptitious reputation. Little is positively done by such bodies;—those who are truly capable, have generally reached that period of life when repose brings with it more attractions than labour, and the mere charlatans in science are as useless occupants of the places of the Academy, as of the chairs of the School of Medicine.

